# Continuous Treatment with Tofacitinib but Not Filgotinib Is Effective in Non-Responders with Active Ulcerative Colitis: A Propensity Score-Matching Analysis

**DOI:** 10.3390/jcm14010217

**Published:** 2025-01-02

**Authors:** Soichi Yagi, Hirokazu Fukui, Maiko Ikenouchi, Tetsuya Shiraishi, Koji Kaku, Midori Wakita, Yasuhiro Takagi, Toshiyuki Sato, Mikio Kawai, Koji Kamikozuru, Yoko Yokoyama, Tetsuya Takagawa, Toshihiko Tomita, Shinichiro Shinzaki

**Affiliations:** 1 Department of Gastroenterology, Faculty of Medicine, Hyogo Medical University, 1-1 Mukogawa, Nishinomiya 663-8501, Hyogo, Japan; so-yagi@hyo-med.ac.jp (S.Y.); hfukui@hyo-med.ac.jp (H.F.); ma-ikenouchi@hyo-med.ac.jp (M.I.); te-shiraishi@hyo-med.ac.jp (T.S.); ds23003@hyo-med.ac.jp (K.K.); mi-wakita@hyo-med.ac.jp (M.W.); ya-takagi@hyo-med.ac.jp (Y.T.); tshnngn@hyo-med.ac.jp (T.S.); mkawai@hyo-med.ac.jp (M.K.); turuturu@hyo-med.ac.jp (K.K.); yoko0502@hyo-med.ac.jp (Y.Y.); tomita@hyo-med.ac.jp (T.T.); 2 Center for Clinical Research and Education, Hyogo Medical University, Nishinomiya 663-8501, Hyogo, Japan; takagawa@hyo-med.ac.jp

**Keywords:** inflammatory bowel disease, ulcerative colitis, JAK inhibitor, tofacitinib, filgotinib

## Abstract

**Background:** Few studies have compared the efficacy and safety of Janus kinase (JAK) inhibitors in patients with ulcerative colitis (UC). We compared the real-world effectiveness and safety of tofacitinib (TOF) and filgotinib (FIL) as induction therapy for UC by propensity score-matching analysis. **Methods:** We enrolled 230 patients with active UC who received either TOF (n = 197) or FIL (n = 33) as induction therapy. The primary outcome was the clinical response at week 8, and the secondary outcomes were the clinical response/remission rates from weeks 2–8, including the course of patients without a clinical response/remission at week 4. **Results:** Propensity score-matching analysis revealed that the clinical response rate gradually increased to 72.2% at 8 weeks in the TOF group, whereas it tended to decrease to 48.5% in the FIL group. Clinical remission rates increased from 2 (36.7% vs. 36.7%) to 8 weeks (63.6% vs. 48.5%) after treatment in the TOF and FIL groups, respectively. The clinical response rate was higher in the TOF group than in the FIL group at week 8 in patients without a clinical response at week 4 (38.5% vs. 0%; *p* = 0.011). The clinical remission rate was also higher in the TOF group than in the FIL group at week 8 in patients without clinical remission at week 4 (50.0% vs. 16.7%; *p* = 0.046). The incident rates of infection and anemia were higher in the TOF group than in the FIL group. **Conclusions:** TOF may be more effective than FIL at 8 weeks for patients with UC who do not respond to treatment within the first 4 weeks.

## 1. Introduction

Ulcerative colitis (UC) is an inflammatory bowel disease phenotype characterized by chronic diarrhea, bloody stools and abdominal pain [1]. Although the patients with UC had been treated with 5-aminosalicylic acid and steroid, its treatment has greatly advanced by the development of anti-tumor necrosis factor α antibody (aTNF) in the early 2000s. However, there are considerable number of UC patients who are refractory to not only steroid but also aTNF. For these patients, a variety of medicines with different action mechanism, such as adhesion molecule inhibitors and interleukin-12/23 inhibitors, have been developed and used to treat patients with UC. Furthermore, Janus kinase (JAK) inhibitors that had been used to treat various immunological diseases such as rheumatoid arthritis and psoriasis [2,3] are currently available as a new therapeutic option for UC patients. Indeed, some placebo-controlled randomized clinical trials and real-world studies have revealed that JAK inhibitors are very useful for treating patients with UC [4,5]. However, there is limited studies comparing the efficacy among JAK inhibitors, and therefore, such studies are urgently needed to accumulate more clinical evidence for patients treated with JAK inhibitors.

JAK is a tyrosine kinase that binds to the intracytoplasmic domain of cytokine receptors. Cytokine stimulation activates JAKs, which then promote the phosphorylation of transcriptional factors such as signal transducers and activators of transcription, leading to enhanced target gene expression [6]. JAKs consist of four isoforms such as JAK1, JAK2, JAK3 and tyrosine kinase 2 [7], and each JAK isoform is likely to show distinct functional implications. For instance, JAK1 and JAK3 are mainly associated with lymphocyte signaling under inflammatory condition, while JAK2 is involved in the proliferation and/or differentiation of hematopoietic cells [8]. In this context, it may be interesting to speculate that the different selectivity of JAK inhibitors may affect the clinical outcomes and/or safety. A phase III clinical trial confirmed that tofacitinib (TOF; JAK 1–3 inhibitor) is effective for induction/maintenance therapy in patients with active UC [4]. In detail, this study demonstrated that the rate of clinical remission was 18.5% at 8 weeks after TOF treatment [4]. Subsequently, TOF was first approved for treating adult patients with active UC in Japan in 2018. We previously reported that TOF is effective for patients with UC, regardless of previous treatment [9]. This remarkable efficacy of TOF may depend on wide-ranged inhibition of JAK 1–3. Thereafter, a phase IIb/III clinical trial demonstrated that filgotinib (FIL; JAK 1 pre-dominant inhibitor) is also effective for induction/maintenance therapy in UC patients [5], and it was approved for treating patients with UC in Japan in 2022. Moreover, Danese et al. have recently demonstrated in phase III trial that upadacitinib (UPA; JAK 1 pre-dominant inhibitor) is effective for induction/maintenance therapy in UC patients [10], resulting in the approval of use of UPA in the treatment of UC in Japan. Therefore, the data of TOF has been well accumulated, whereas those of FIL and UPA is just accumulating and the comparative analysis between TOF and other JAK inhibitors are now highlighted. JAK inhibitors are usually applied to the patients with active UC who are refractory to aTNF, and they are being used frequently. Therefore, in order to determine whether clinical physicians should continue or suspend JAK inhibitors treatment, they urgently wish to know as possible as early whether the applied JAK inhibitors are likely to bring a clinical response/remission in their UC patients. On the other hand, adverse events are also important factors to determine the therapeutic strategy. Thus, comparative information among JAK inhibitors is strongly needed to decide which JAK inhibitor is best suited for different cases of UC; however, such comparative data is relatively lacking. In the present study, we therefore compared the rapid- and short-term efficacy and safety of TOF and FIL as induction therapy in patients with UC.

## 2. Materials and Methods

### 2.1. Study Design and Participants

To compare the efficacy and safety of TOF and FIL for patients with UC, this retrospective observational study was conducted at Hyogo Medical University Hospital. The study was performed with approval (No. 4263) from the Ethics Committee of Hyogo Medical University and conducted according to the principles governing human research stipulated by the Declaration of Helsinki. Patients diagnosed with UC on the basis of clinico-endoscopic evidence were included. Efficacies of TOF and FIL were evaluated based on the partial Mayo score (PMS). We reviewed the medical records of all patients with UC who received TOF or FIL treatment as induction therapy between May 2018 and October 2023. Among them, we isolated patients with active UC matching the following criteria: PMS ≥ 3 or PMS = 2 and a rectal bleeding subscore ≥ 1 [11]. Patients who did not satisfy the above conditions were excluded from the analysis. In addition, patients with the following conditions were excluded: history of surgery or follow-up period <8 weeks. All patients included in the analysis received TOF (10 mg) twice daily or FIL (200 mg) once daily for 8 weeks from the start of treatment.

### 2.2. Outcomes and Definitions

The primary outcome was clinical response at week 8 after the administration of TOF or FIL. The clinical response was defined as follows: a decrease in the PMS from baseline ≥ 2 and a decrease in the rectal bleeding subscore ≥ 1 or a rectal bleeding subscore ≤ 1. Clinical remission was defined as follows: PMS ≤ 2, each subscore ≤ 1 and a rectal bleeding subscore of 0.

In the clinical setting, the information regarding rapid efficacy of JAK inhibitors is needed to decide whether the treatment should be exchanged. Therefore, we determined the secondary outcomes as follows: the clinical response and remission from weeks 2 to 8, including the course of patients without clinical response/remission at week 4 after beginning TOF or FIL administration, and the safety during the observation period. The reported safety outcomes included the number of serious adverse events, adverse events leading to discontinuation, infections, and abnormal laboratory values per 100 patient-years. The severity of adverse events and abnormal laboratory values was graded using the modified Common Terminology Criteria for Adverse Events, version 5.0.

### 2.3. Statistical Analysis

Continuous variables are expressed as medians with ranges. Categorical data were compared using Fisher’s exact test, and continuous data were compared using the Mann-Whitney *U* test. To minimize bias when comparing each group, propensity score-matching was conducted. The following variables that may affect the efficacy were selected: age, sex, disease duration, PMS, and baseline serum albumin and C-reactive protein levels. After matching, the clinical outcomes were compared between the matched TOF and FIL groups. The exposure-adjusted incidence rate (per 100 patient-years) was defined as the number of patients who experienced an adverse event divided by the total exposure time among the patients. A *p* value < 0.05 was considered statistically significant. All statistical analyses were performed using EZR Version 1.61 (Saitama Medical Centre, Jichi Medical University).

## 3. Results

### 3.1. Patient Characteristics

**Figure 1:**
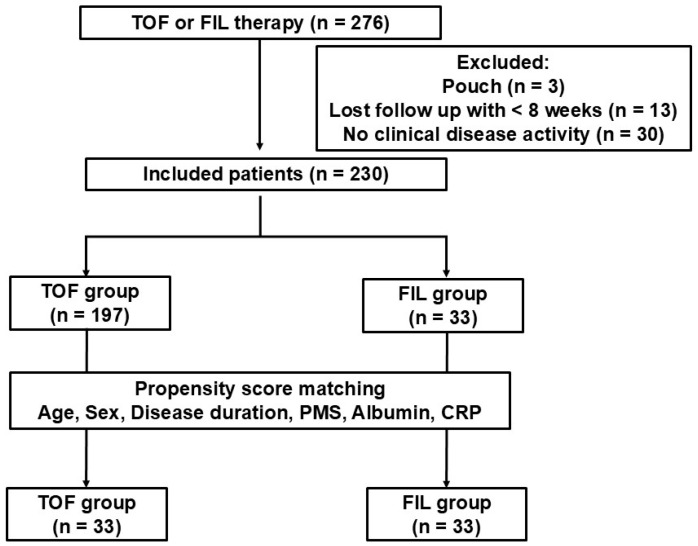
Flowchart of patients with UC included in the analysis. Patients were treated with tofacitinib (TOF, 10 mg twice daily) or filgotinib (FIL, 200 mg). UC, ulcerative colitis; TOF, tofacitinib; FIL, filgotinib; PMS, partial Mayo score; CRP, C-reactive protein.

A flowchart of this study is shown in Figure 1. We first enrolled a total of 276 patients with UC who were initiating treatment with TOF or FIL. After excluding 46 patients, 197 TOF-treated and 33 FIL-treated patients were analyzed. Baseline characteristics of patients in the TOF and FIL groups are shown in Table 1. Significant differences between the TOF and FIL groups were detected in the age at induction, as well as the baseline serum albumin, and C-reactive protein levels. In addition, the rate of previous treatment failure of ustekinumab and concomitant medication (5-aminosalicylate and thiopurine) differed between the two groups although some patients were included in more than one of these categories. None of the other clinical and biochemical disease activity variables were significantly different between the TOF and FIL groups.

### 3.2. Efficacy Outcomes

**Figure 2:**
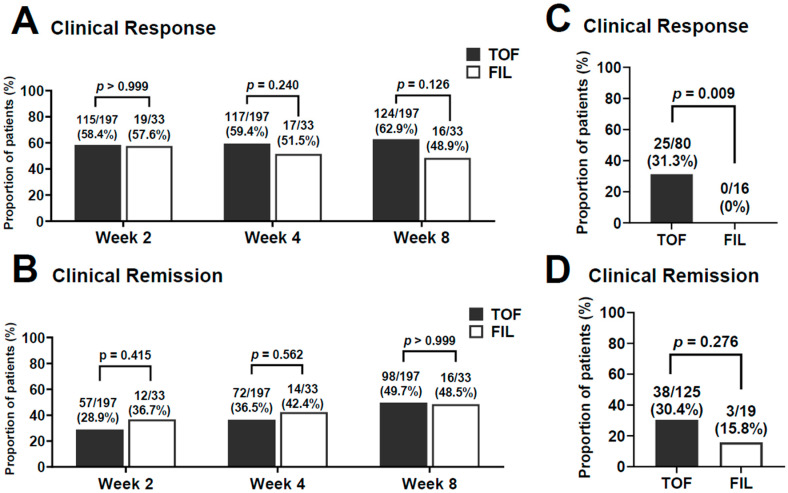
Time course of the effect of tofacitinib (TOF) and filgotinib (FIL) for patients with active UC. (**A**) Clinical response. (**B**) Clinical remission. Efficacy outcome at week 8 in patients without a clinical response to TOF or FIL treatment at week 4. (**C**) Clinical response. (**D**) Clinical remission.

The clinical response rates at 2 weeks were 58.4% and 57.6% in the TOF and FIL groups, respectively (Figure 2A; *p* > 0.999). Although the clinical response rate did not differ statistically between the two groups at any time point, the maximum clinical response rates in the TOF and FIL groups were 62.9% at 8 weeks and 51.5% at 4 weeks after induction, respectively. The clinical remission rates were increased at 8 weeks in both groups. The maximum clinical remission rates in the TOF and FIL groups were 49.7% and 48.5% at 8 weeks after induction and did not significantly differ between groups at any time point (Figure 2B).

At 4 weeks after induction, there were 80 non-responders (40.6%) in the TOF group and 16 (48.5%) in the FIL group (Figure 2C). The number of non-responders that became responders at 8 weeks after induction was significantly higher in the TOF group (31.3%) than in the FIL group (0%) (*p* = 0.009). At 4 weeks after induction, 125 patients (63.5%) in the TOF group and 19 (57.6%) in the FIL group did not achieve remission (Figure 2D). Among these patients, at 8 weeks after induction, 38 patients (30.4%) in the TOF group, and 3 patients (15.8%) in the FIL group did achieved remission (*p* = 0.276).

### 3.3. Comparison of Efficacy Outcome After Propensity Score-Matching

Thirty-three patients in both the TOF and FIL groups were isolated using propensity score-matching. The characteristics of the isolated patients are summarized in Table 2. Propensity score-matching revealed no significant differences in the baseline characteristics between the TOF and FIL groups.

**Figure 3:**
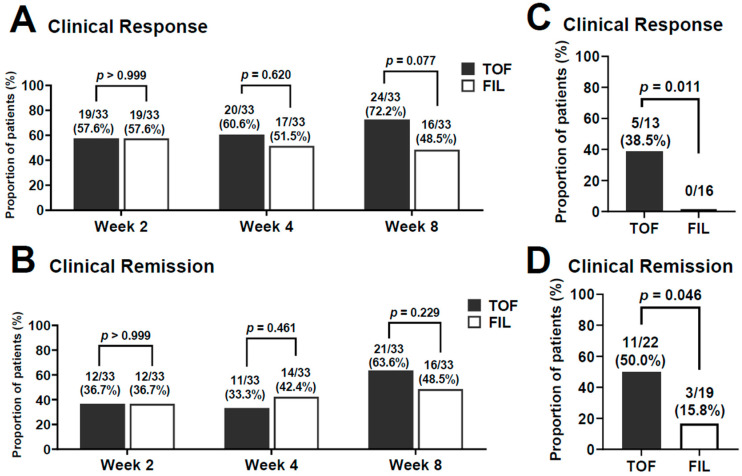
Time course of the effect of tofacitinib (TOF) and filgotinib (FIL) for patients with active UC after propensity score-matching. (**A**) Clinical response. (**B**) Clinical remission. Propensity score-matching analysis showing the efficacy outcome at week 8 in patients with no response to TOF or FIL treatment at week 4. (**C**) Clinical response. (**D**) Clinical remission.

The clinical response and remission rates after propensity score-matching are shown in Figure 3. The clinical response rates at 2 weeks were 57.6% and 57.6% in the TOF and FIL groups, respectively (Figure 3A). Thereafter, the clinical response rate gradually increased to 72.2% at 8 weeks in the TOF group, whereas it tended to decrease to 48.5% at 8 weeks in the FIL group although there was no significant difference between the two groups at each time point. On the other hand, the clinical remission rate increased higher from 2 (36.7% vs. 36.7%) to 8 weeks (63.6% vs. 48.5%) after treatment in the TOF and FIL groups, respectively (Figure 3B). No significant difference in the clinical remission rates at each time point was detected between the two groups.

We also investigated non-responders at 4 weeks after induction of TOF or FIL treatment (Figure 3C,D). Notably, at 8 weeks after induction, 5 of 13 non-responders (38.5%) in the TOF group became responders, whereas none of the 16 non-responders in the FIL group became responders. The clinical response rate was significantly higher in the TOF group compared with the FIL group (*p* = 0.011). Remission was achieved at 8 weeks after induction in 11 of 22 non-remitted patients (50.0%) in the TOF group and in 3 of 19 (15.8%) in the FIL group. The clinical remission rate was significantly higher in the TOF group (*p* = 0.046).

### 3.4. Safety Outcome

The numbers of adverse events reported during the observational period are presented in Table 3. None of the patients in either group died or had life-threatening events. Adverse events occurred in 106 of 197 (53.8%) of patients in the TOF group and 11 of 33 (33.3%) in the FIL group. The highest adverse event with the highest rate was hypertriglyceridemia in both the TOF (19.2/100 patient-years) and FIL (17.4/100 patient-years) groups. The incidence rates of upper respiratory symptoms (5.8/100 patient-years), herpes zoster infection (3.1/100 patient-years) and anemia (14.9/100 patient-years) were higher in the TOF group than in the FIL group.

## 4. Discussion

JAK inhibitors have been applied for the treatment of patients with active UC [4,5]. Moreover, JAK inhibitors are now an option for the treatment of patients with UC who are refractory to treatment with anti-tumor necrosis factor-alpha agents [12]. Thus, the use of a JAK inhibitor as a second-line-advanced therapy in patients with UC has become more common. However, clinical evidences supporting the efficacy of JAK inhibitors for UC remains limited. In the present study, we investigated the efficacy of TOF and FIL for patients with UC during the treatment time course and evaluated differences between TOF and FIL to establish a strategy for the appropriate use of JAK inhibitors for treating UC. As a whole, approximal 30% of patients with UC achieved clinical remission after 2 weeks of treatment in both the TOF and FIL groups. Because the patients investigated were classified as having active UC, this high rate in the early phase seems to be significant in the clinical setting. At 8 weeks after treatment with TOF or FIL, the clinical remission rates were approximately 50% in both groups, suggesting that TOF and FIL have equivalent efficacy to achieve clinical remission in overall patients with active UC. However, a considerable number of non-responders at 4 weeks after induction of TOF treatment (31.3%) became responders at 8 weeks after treatment although none of the non-responders after 4-weeks of FIL treatment became responders at the 8-week point. These findings suggest that TOF treatment should be continued even if the patients do not show a significant clinical response at 4 weeks after induction. In contrast, patients who did not show a clinical response to FIL treatment at 4 weeks after induction might not expect a significant clinical response even if they receive continuous FIL treatment for up to 8 weeks after induction. Because these findings were based on an unbalanced number of patients with differing characteristics, we reassessed our data using a propensity score-matching analysis.

While clinical retrospective studies are useful for obtaining important information in actual clinical practice, they are inherently susceptible to biases, such as missing data and inclusion biases. A propensity score-matching analysis is frequently performed to limit systematic differences in patients’ baseline characteristics, reducing the effects of confounders and multiple covariates to afford comparability [13]. In this context, we used a propensity score-matching analysis to reassess and compare the efficacies of TOF and FIL over the course of treatment. Previous studies have reported that the clinical response rate at 8 weeks after TOF treatment is approximately 60% [4,14]. On the other hand, its rate at 10 weeks after FIL treatment is approximately ranged 40–60% [5,15,16]. These results seem to be compatible with our obtained data in the present study, Thus, the clinical response rate tended to be higher in the TOF group (72.2%) than in the FIL group (48.5%) at week 8 although no statistical difference was detectable. As for clinical remission rates, previous studies have reported that the clinical remission rate at 8 weeks after TOF treatment is approximately ranged 20–40% [4,11,14], whereas its rate at 10 weeks after FIL treatment is approximately ranged 20–50% [5,15,16]. Although these results also compatible with our obtained data in the present study, the propensity score-matching analysis additionally revealed interesting findings. Of note, the clinical response and remission rates were significantly greater in non-responders in the TOF group than in the FIL group at week 4. These findings suggest that TOF treatment should be continued even if patients do not show a significant clinical response at 4 weeks after induction.

TOF and FIL may exhibit different behaviors to achieve their effects on the clinical outcome. While we currently lack an exact explanation, the differences in their specific target molecules may contribute to the differences in the time-course of their effectiveness. TOF inhibits all JAKs but preferentially inhibits JAK1 and JAK3 [17,18]. On the other hand, FIL is presumed to be a selective JAK1 inhibitor [19]. This may explain the higher prevalence of infections in the TOF group compared with the FIL group, because broad inhibition of JAKs is associated with strong immunosuppression, resulting in a risk of infection [20,21]. Indeed, it is very difficult to compare the efficacy and safety of TOF and FIL treatment as the metabolic characteristics and administered dosages are quite different. For example, the half-life of TOF is 3 h [22], whereas that of FIL is 6 h for the parent compound and 23 h for the active metabolite [23]. In addition, in the present study, TOF was administered a dose of 10 mg twice daily and FIL was administered at a dose of 200 mg once daily for induction therapy; therefore, different efficacies due to differences in the administration methods cannot be ruled out.

The metabolic pathways of TOF and FIL also differ. In detail, TOF is primarily metabolized by cytochrome P450 (CYP), while FIL is mainly metabolized by carboxylesterase 2 [24]. There are more than 2000 possible mutations of CYP and some single nucleotide polymorphisms have a large impact on CYP activity [25]. On the other hand, accumulating evidence suggests that many factors, including genetic polymorphisms, drug-drug interactions, and drug-disease interactions, have an important role in determining the variability in the therapeutic response to carboxylesterase-substrate drugs [26]. Although we have no genetic data for our enrolled patients enrolled, an analysis of genetic alterations related to drug metabolism and clinical outcomes could provide interesting information.

Adverse events are important factors to determine the therapeutic strategy. In the present study, we found that the incident rates of infection and anemia were higher in the TOF group than in the FIL group. This might be due to the difference of selectivity of JAK inhibitions. TOF is likely to inhibit all JAKs whereas FIL selectively inhibit JAK1, possibly resulting in higher rates of adverse events in TOF group. Although we might emphasize the efficacy of TOF in the treatment of UC, the use of FIL may become an important option for patients who hesitate TOF treatment due to its adverse events.

TOF is the first JAK inhibitor approved for the treatment of patients with UC. Therefore, several clinical trials have revealed that TOF is significantly effective in long-term (clinical remission rate, 35–45% after 52 weeks treatment) in patients with UC [4,11,27]. Regarding FIL, a few papers have reported that FIL is also significantly effective in long-term (clinical remission rate, 37–60% after 58 weeks treatment) in patients with UC [5,15] although accumulating evidences are still required. In the present study, we focused rapid- and short-term effect of TOF and FIL in the treatment of UC and suggested that the response at week 4 is a key to predict the later clinical outcome. Thus, the non-responder at week 4 in TOF group still have a possibility to become a responder by additional 4-weeks treatment, whereas those in FIL group hardly have such possibility. Therefore, the validity of continuous treatment with FIL may be determined at week 4. On the other hand, TOF is recommended to be reduced the dosage from 20 mg/day to 10 mg/day after 8-weeks treatment because its long-term use is likely to cause cardiac events, venous thromboembolism or infection. Subsequently, the reduction of TOF is associated with high-risk of relapse, and high-dose of TOF is continuously used for patients with intractable UC in the actual clinical setting, concerning the higher risk of severe adverse events. In this context, since the severity of adverse events in FIL treatment appears to be lower than that in TOF treatment [28], it may be an interesting idea that TOF treatment is switched to FIL treatment at the timing of TOF dosage reduction. At present, we have no data regarding the switch from some JAK inhibitor to another one, and this issue may be highlighted in a near future. On the other hand, the prevalence of older UC patients is increasing in the world, implying the importance of adverse events rather than effectiveness in those patients. From this viewpoint, FIL may be suitable for older UC patients rather than TOF although this issue may also be highlighted in a near future.

Our study has several limitations. First, it was a single-center retrospective study involving a relatively small number of patients, and we did not measure other possible confounders such as concomitant drugs. Further, we did not compare the efficacy of TOF and FIL with upadacitinib, which was approved 6 months after FIL. Upadacitinib may have a distinct role compared with TOF and FIL, warranting a more detailed and separate investigation.

In summary, this is the first real-world study to compare the short-term outcomes of TOF and FIL treatment for patients with UC in a time-dependent manner. Our findings demonstrated that clinical response and remission rates at 8 weeks were significantly greater in patients who were non-responders at week 4 in the TOF group than in those in the FIL group, suggesting that TOF treatment should be continued even if the patients do not show a significant clinical response at 4 weeks after induction. In contrast, as the effect of FIL seems to reach a plateau at 4 weeks, patients who do not respond by this time point may benefit more from alternative treatments. Concerning the adverse events, the incident rates of infection and anemia were higher in the TOF group than in the FIL group, suggesting that FIL may be safer for patients with bone marrow suppression and/or high susceptibility to infection. Although further prospective multicenter studies are necessary, we believe that our first real-world study will be useful for planning strategies using JAK inhibitors for the treatment of UC in the clinical setting.

## Figures and Tables

**Table 1:** Baseline characteristics of patients.

	Tofacitinib (n = 197)	Filgotinib (n = 33)	*p* Value
Age, y	37 (13–87)	49 (15–71)	0.035
Female	75 (38.1)	11 (33.3)	0.74
UC duration, y	3 (0–42)	5 (0–31)	0.25
Extent of disease			
Pancolitis	158 (80.2)	29 (87.9)	0.35
Left-sided colitis	36 (18.3)	3 (9.1)	0.31
Proctitis	3 (1.5)	1 (3.0)	0.46
Partial Mayo Score	6 (2–9)	5 (2–8)	0.18
Laboratory examination			
Hemoglobin, g/dL	12.6 (6.7–16.8)	13.2 (10.1–16.2)	0.26
Albumin, g/dL	3.8 (2.1–6.4)	4.0 (3.3–4.9)	0.031
C-reactive protein, mg/dL	0.38 (0–8.26)	0.18 (0.01–3.15)	0.008
Previous treatment failure			
Corticosteroid	186 (94.4)	28 (84.8)	0.061
TNF antagonist	109 (55.3)	13 (39.3)	0.094
Vedolizumab	31 (15.7)	7 (21.2)	0.45
Ustekinumab	14 (7.1)	9 (27.2)	0.002
Concomitant therapy at induction			
5-Aminosalicylate	123 (62.4)	27 (81.8)	0.031
Thiopurine	0	8 (24.2)	<0.001
Corticosteroid	38 (19.3)	5 (15.2)	0.81

Values are presented as the median (range) or n (%). UC, ulcerative colitis; TNF, tumor necrosis factor.

**Table 2:** Clinical characteristics after propensity score-matching.

	Tofacitinib (n = 33)	Filgotinib (n = 33)	*p* Value
Age, y	46 (18–67)	49 (15–71)	0.96
Female	12	11	1
UC duration, y	5 (0–20)	5 (0–31)	0.83
Partial Mayo Score	5 (3–8)	5 (2–8)	0.88
Laboratory examination			
Albumin, g/dL	4.1 (2.7–4.6)	4.0 (3.3–4.9)	0.81
C-reactive protein, mg/dL	0.16 (0–1.68)	0.18 (0.01–3.15)	0.74

Values are presented as the median (range). UC, ulcerative colitis.

**Table 3:** Adverse events.

	Tofacitinib (n = 197)		Filgotinib (n = 33)	
	n	n/100 Patient-Years	n	n/100 Patient-Years
Serious adverse events	0	0	0	0
Adverse events leading to discontinuation	15	8.0	2	6.4
Infections	22	12.1	1	3.1
Upper respiratory	11	5.8	0	0
Herpes labialis	1	1	1	3.1
Herpes zoster	6	3.1	0	0
Abnormal laboratory test results				
Anemia	26	14.9	1	3.1
Leukopenia	4	2.1	1	3.1
Hypoalbuminemia	8	4.2	0	0
Elevated serum amylase levels	3	1.5	3	9.9
Hypercholesterolemia	2	1.5	0	0
Hypertriglyceridemia	17	19.2	6	17.4

## Data Availability

The data underlying this article will be shared upon reasonable request to the corresponding author.

## References

[1] Ungaro R., Mehandru S., Allen P.B., Peyrin-Biroulet L., Colombel J.F. (2017). Ulcerative colitis. Lancet.

[2] Lee Y.H., Song G.G. (2020). Comparative efficacy and safety of tofacitinib, baricitinib, upadacitinib, filgotinib and peficitinib as monotherapy for active rheumatoid arthritis. J. Clin. Pharm. Ther..

[3] Kvist-Hansen A., Hansen P.R., Skov L. (2020). Systemic treatment of psoriasis with JAK inhibitors: A review. Dermatol. Ther..

[4] Sandborn W.J., Su C., Sands B.E., D’Haens G.R., Vermeire S., Schreiber S., Danese S., Feagan B.G., Reinisch W., Niezychowski W. (2017). Tofacitinib as induction and maintenance therapy for ulcerative colitis. N. Engl. J. Med..

[5] Feagan B.G., Danese S., Loftus E.V., Vermeire S., Schreiber S., Ritter T., Fogel R., Mehta R., Nijhawan S., Kempiński R. (2021). Filgotinib as induction and maintenance therapy for ulcerative colitis (selection): A phase 2b/3 double-blind, randomised, placebo-controlled trial. Lancet.

[6] Salas A., Hernandez-Rocha C., Duijvestein M., Faubion W., McGovern D., Vermeire S., Vetrano S., Casteele N.S. (2020). Jak–STAT pathway targeting for the treatment of inflammatory bowel disease. Nat. Rev. Gastroenterol. Hepatol..

[7] Villarino A.V., Kanno Y., O’Shea J.J. (2017). Mechanisms and consequences of Jak-STAT signaling in the immune system. Nat. Immunol..

[8] Ghoreschi K., Laurence A., O’Shea J.J. (2009). Janus kinases in immune cell signaling. Immunol. Rev..

[9] Kojima K., Watanabe K., Kawai M., Yagi S., Kaku K., Ikenouchi M., Sato T., Kamikozuru K., Yokoyama Y., Takagawa T. (2024). Real-world efficacy and safety of tofacitinib treatment in Asian patients with ulcerative colitis. World J. Gastroenterol..

[10] Danese S., Vermeire S., Zhou W., Pangan A.L., Siffledeen J., Greenbloom S., Hébuterne X., D’Haens G., Nakase H., Panés J. (2022). Upadacitinib as induction and maintenance therapy for moderately to severely active ulcerative colitis: Results from three phase 3, multicentre, double-blind, randomised trials. Lancet.

[11] Chaparro M., Acosta D., Rodríguez C., Mesonero F., Vicuña M., Acosta M.B., Fernández-Clotet A., Martínez Á., Arroyo M., Vera I. (2023). Real-world evidence of tofacitinib in ulcerative colitis: Short-term and long-term effectiveness and safety. Am. J. Gastroenterol..

[12] Tessa S., Biemans V.B.C., Marijn V., Frank H., Hoentjen F., Vries A., Bodegraven A.A., Bodelier A., Boer N.K.H., Dijkstra G. (2023). Superior effectiveness of tofacitinib compared to vedolizumab in anti-tnf-experienced ulcerative colitis patients: A nationwide dutch registry study. Clin. Gastroenterol. Hepatol..

[13] Corrigan-Curay J., Sacks L., Woodcock J. (2018). Real-world evidence and real-world data for evaluating drug safety and effectiveness. JAMA.

[14] Chaparro M., Garre A., Mesonero F., Rodríguez C., Acosta M.B., Martínez-Cadilla J., Arroyo M., Manceñido N., Sierra-Ausín M., Vera-Mendoza I. (2021). Tofacitinib in ulcerative colitis: Real-world evidence from the ENEIDA registry. J. Crohns Colitis..

[15] Akiyama S., Yokoyama K., Yagi S., Shinichiro S., Tsuruta K., Yoshioka S., Sako M., Shimizu H., Kobayashi M., Sakurai T. (2024). Efficacy and safety filgotinib for ulcerative colitis: A real-world multicenter retrospective study in Japan. Aliment. Pharmacol. Ther..

[16] Genovese M.C., Kalunian K., Gottenberg J., Mozaffarian N., Bartok B., Matzkies F., Gao J., Guo Y., Tasset C., Sundy J.S. (2019). Effect of filgotinib vs placebo on clinical response in patients with moderate to severe rheumatoid arthritis refractory to disease-modifying antirheumatic drug therapy. The FINCH 2 Randomized Clinical Trial. JAMA.

[17] Danese S., Grisham M., Hodge J., Telliez J.B. (2016). Jak inhibition using tofacitinib for inflammatory bowel disease treatment: A hub for multiple inflammatory cytokines. Am. J. Physiol. Gastrointest. Liver Physiol..

[18] Flanagan M.E., Blumenkopf T.A., Brissette W.H., Brown M.F., Casavant J.M., Shang-Poa C., Doty J.L., Elliott E.A., Fisher M.B., Hines M. (2010). Discovery of CP-690,550: A potent and selective janus kinase (JAK) inhibitor for the treatment of autoimmune diseases and organ transplant rejection. J. Med. Chem..

[19] Van Rompaey L., Galien R., van der Aar E.M., Clement-Lacroix P., Nelles L., Smets B., Lepescheux L., Christophe T., Conrath K., Vandeghinste N. (2013). Preclinical characterization of GLPG0634, a selective inhibitor of JAK1, for the treatment of inflammatory diseases. J. Immunol..

[20] Din S., Selinger C.P., Black C.J., Ford A.C. (2023). Systematic review with network meta-analysis: Risk of herpes zoster with biological therapies and small molecules in inflammatory bowel disease. Aliment. Pharmacol. Ther..

[21] Clarke B., Yates M., Adas M., Bechman K., Galloway J. (2021). The safety of JAK-1 inhibitors. Rheumatology.

[22] Ma G., Xie R., Strober B., Langley R., Ito K., Krishnaswami S., Wolk R., Valdez H., Rottinghaus S., Tallman A. (2018). Pharmacokinetic characteristics of tofacitinib in adult patients with moderate to severe chronic plaque psoriasis. Clin. Pharmacol. Drug Dev..

[23] Namour F., Diderichsen P.M., Cox E., Vayssière B., Ver der Aa A., Tasset C., Klooster G.V. (2015). Pharmacokinetics and pharmacokinetic/pharmacodynamic modeling of filgotinib (GLPG0634), a selective JAK1 inhibitor, in support of phase iib dose selection. Clin. Pharmacokinet..

[24] Taylor P.C., Choy E., Baraliakos X., Szekanecz Z., Xavier R.M., Isaacs J.D., Strengholt S., Parmentier J.M., Lippe R., Tanaka Y. (2024). Differential properties of janus kinase inhibitors in the treatment of immune-mediated inflammatory diseases. Rheumatology.

[25] Preissner S.C., Hoffmann M.F., Preissner R., Dunkel M., Gewiess A., Preissner S. (2013). Polymorphic cytochrome P450 enzymes (CYPs) and their role in personalized therapy. PLoS ONE.

[26] Laizure S.C., Herring V., Hu Z., Witbrodt K., Parker R.B. (2013). The role of human carboxylesterases in drug metabolism: Have we overlooked their importance?. Pharmacotherapy.

[27] Sandborn W.J., Peyrin-Biroulet L., Quirk D., Wang W., Nduaka C.I., Mukherjee A., Su C., Sands B.E. (2022). Efficacy and safety of extended induction with tofacitinib for the treatment of ulcerative colitis. Clin. Gastroenterol. Hepatol..

[28] Olivera P.A., Lasa J.S., Bonovas S., Danese S., Peyrin-Biroulet L. (2020). Safety of janus kinase inhibitors in patients with inflammatory bowel diseases or other immune-mediated diseases: A systematic review and meta-analysis. Gastroenterology.

